# Heparin-induced thrombocytopenia during anticoagulation therapy for COVID-19-related pulmonary embolism: A case report

**DOI:** 10.1097/MD.0000000000040732

**Published:** 2024-12-06

**Authors:** Yu Zhang, Zhenling Chen, Jianying Li, Xuejing Wang, Yi Liu

**Affiliations:** a Civil Aviation General Hospital, Beijing, China.

**Keywords:** anticoagulation, case report, COVID-19, heparin-induced thrombocytopenia, pulmonary embolism

## Abstract

**Rationale:**

One of the main characteristics of COVID-19 is the high incidence of venous thromboembolism, particularly pulmonary embolism. Anticoagulation therapy is the primary treatment for pulmonary embolism. Heparin-induced thrombocytopenia (HIT) is an antibody-mediated adverse reaction to heparin that occurs during its use of heparin drugs. The main clinical manifestation is a decrease in platelet count, which can lead to the formation of arterial and venous thrombosis and, in severe cases, even death. Herein, we present a case of HIT that occurred during anticoagulation therapy for COVID-19, complicated by pulmonary embolism.

**Patient concerns:**

An 86-year-old man with COVID-19 experienced a significant decrease in platelet count and progression of venous thrombosis in the lower extremities during anticoagulation therapy with nadroparin.

**Diagnoses:**

The 4T score was 6; therefore, HIT was considered.

**Interventions and outcomes:**

All heparin-based drugs were discontinued, and argatroban was administered as anticoagulation therapy. The patient’s platelet count was monitored, and it gradually returned to normal.

**Lessons:**

Clinicians should remain vigilant to venous thromboembolism for COVID-19 patients even after recovery. During anticoagulant therapy, if thrombocytopenia occurs, HIT should be considered due to its high mortality rate. The 4T scoring system was used for the initial assessment. HIT antibodies can be detected, if necessary, to assist in diagnosis and reduce the occurrence of severe HIT. In the future, by detecting certain biomarkers, we can screen out patients with HIT who are more prone to thrombotic events, thereby minimizing the risk of bleeding caused by anticoagulation.

## 1. Introduction

Since the first outbreak of COVID-19 at the end of 2019, multiple waves of different strains of the virus have spread worldwide. By January 2023, COVID-19 had caused approximately 6681,433 deaths globally.^[1]^ One of the primary characteristics of COVID-19 is the high incidence of venous thromboembolism (VTE), particularly pulmonary thromboembolism.^[2,3]^ During the early stages of the pandemic, autopsies conducted on COVID-19 patients revealed the presence of numerous microthrombi in the bodies of most patients.^[4,5]^ Consequently, preventive anticoagulation therapy is often necessary for COVID-19 patients at high risk of thrombosis, while anticoagulation therapy is required for those who have already developed thrombi. Heparin drugs are currently the most commonly used anticoagulants in clinical practice, with low-molecular-weight heparin (LMWH) being widely used because of its fewer adverse reactions and ease of use. Heparin-induced thrombocytopenia (HIT) is an antibody-mediated adverse reaction to heparin that occurs during administration. Clinically, it primarily manifests as a decrease in platelet count, which can lead to the formation of arterial and venous thrombi, and in severe cases, can even result in death.^[6,7]^ The mortality rate of untreated HIT can be as high as 20%.^[8]^ HIT accompanied by thrombosis is referred to as HITT (HIT with thrombosis), whereas HIT without thrombosis is called isolated HIT.

There are currently no definitive data on the incidence of HIT in the context of COVID-19 infection. Rostami et al analyzed 39 relevant articles and included a total of 11,334 patients with severe COVID-19 and HIT, among which only 123 cases of HIT were confirmed.^[1]^ To date, reports of HIT secondary to mild-to-moderate COVID-19 infection have mostly come from Western countries, while reports from developing countries are rare. Owing to the high mortality rate of HIT, clinicians in developing countries should pay increased attention to this population. Here, we report the case of an unvaccinated male patient who developed HIT during nadroparin anticoagulation therapy for pulmonary thromboembolism caused by COVID-19. We summarize the clinical characteristics of this case to raise the awareness and attention of clinicians towards such patients.

## 2. Case presentation

**Figure 1.**
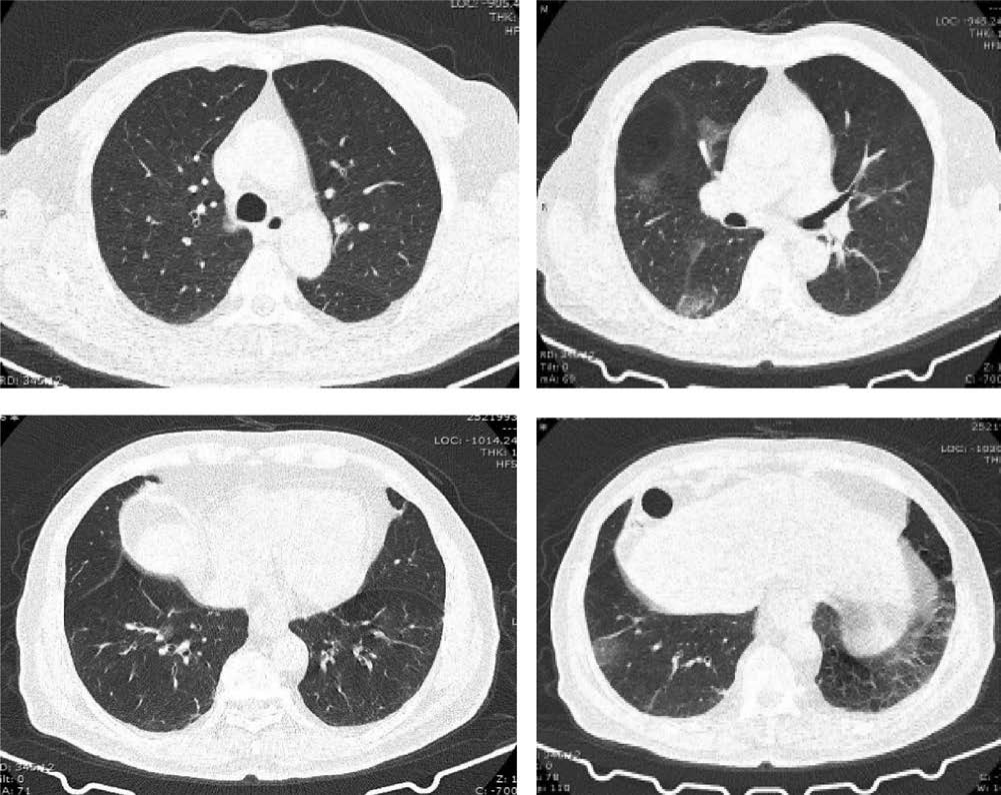
The patient tested positive for COVID-19 antigen, and chest CT showed multiple ground-glass opacities in the right lung.

**Figure 2.**
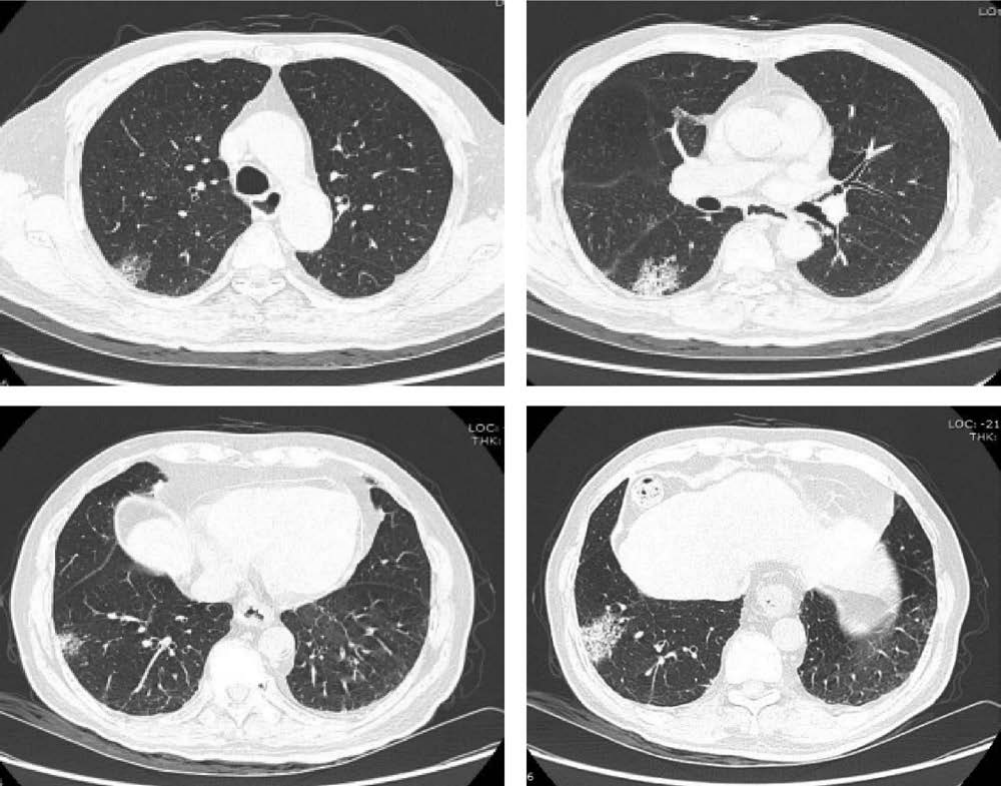
The patient’s COVID-19 antigen and nucleic acid tests turned negative, but a follow-up chest CT scan showed worsening of the lung lesions compared to before.

A male patient, 86 years old, weighing 65 kg, with no underlying health conditions and unvaccinated against COVID-19, presented to our hospital’s fever clinic with fever and cough. The COVID-19 antigen test was positive, and chest CT showed multiple ground-glass opacities in the right lung (Fig. 1). The patient was diagnosed with moderate COVID-19 infection. After antiviral treatment with nirmatrelvir/ritonavir, the patient’s body temperature returned to normal, and follow-up COVID-19 antigen and nucleic acid tests were negative. However, due to unsatisfactory absorption of the lung lesions (Fig. 2), the patient was admitted to our department.

**Figure 3.**
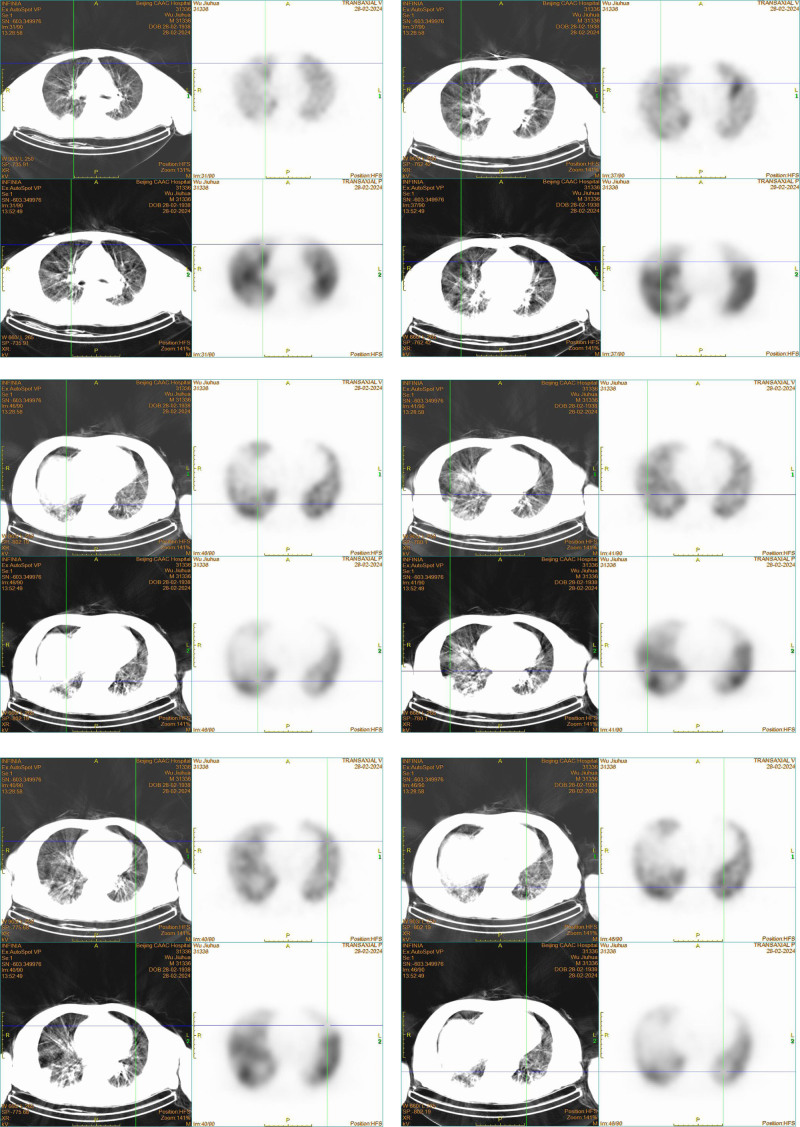
The V/Q scan of the patient indicated multiple pulmonary segmental and subsegmental mismatches between perfusion and ventilation in both lungs, suggesting multiple pulmonary emboli.

Given the higher risk of venous thromboembolism in COVID-19 patients, we initiated prophylactic anticoagulant therapy (Nadroparin 2050 IU QN) immediately after excluding contraindications. Upon admission, further tests revealed a significantly elevated D-dimer level (3.5 mg/L), and a lower-limb ultrasound showed a thrombus in the right calf deep veins. Although the patient did not exhibit symptoms of chest tightness, shortness of breath, or hypoxia and arterial blood gas analysis showed normal oxygen partial pressure without supplemental oxygen, we recommended a CT pulmonary angiogram (CTPA) to rule out pulmonary embolism. Owing to concerns about the impact of contrast agents on renal function, the patient and family chose a ventilation-perfusion (V/Q) scan instead. The V/Q scan results indicated mismatched perfusion and ventilation in multiple lung segments and subsegments, suggesting the presence of multiple pulmonary emboli (Fig. 3). On the 5th day of hospitalization, considering the patient’s significantly elevated D-dimer levels, lower-limb venous thrombosis, and V/Q scan findings, a diagnosis of pulmonary embolism was confirmed. Nadroparin was adjusted to 5600 IU every 12 hours. Two days later, the patient’s peripheral blood routine showed a decrease in platelet count from 182.00 × 10^9^/L to 39.00 × 10^9^/L, and the D-dimer increased to 17.26 mg/L. Follow-up lower-limb ultrasound revealed an increase in thrombi in the deep veins of the right calf.

**Figure 4.**
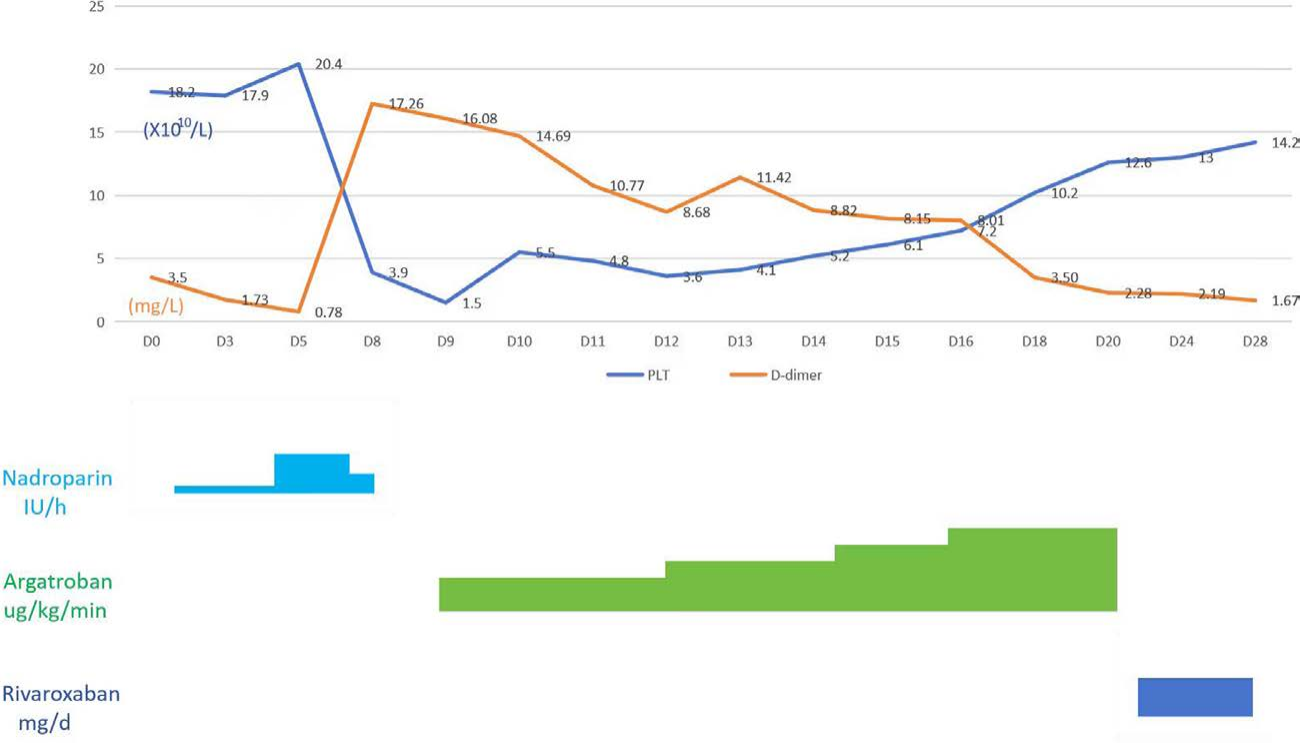
Treatment process and outcome of the patient. Notes: Dosage of nadroparin: 2050IU QD (once daily) from day 1 (D1) to day 4 (D4), 5600IU q12h (every 12 h) from day 5 (D5) to day 7 (D7), 5600IU QD from day 8 (D8). Dosage of argatroban: 0.3ug/kg/min from day 9 (D9) to day 12 (D12), 0.45ug/kg/min from day 13 (D13) to day 15 (D15), 0.6ug/kg/min from day 16 (D16) to day 17 (D17), 0.75ug/kg/min from day 18 (D18) to day 20 (D20). Dosage of rivaroxaban: 15 mg q12h (every 12 h) from day 21 (D21) onwards to the follow-up date. D-dimer = D-dimer concentration (mg/L), PLT = platelet count (×10^9^/L).

Based on the 4T score (which yielded a total of 6 points for this patient as follows: 1 point for platelet count decrease within the range of (10–19) × 10^9^/L; 2 points for the timing of platelet count decrease occurring on the 7th day of nadroparin administration; 1 point for the presence of new thrombosis as indicated by lower extremity ultrasound; and 2 points for the absence of other causes for platelet reduction), a high probability of HIT was considered. Subsequently, nadroparin was discontinued and heparin was removed from the line and replaced with saline flushes. Argatroban was initiated via intravenous infusion for anticoagulation. Given the patient’s advanced age and high bleeding risk, we started with a low-dose of argatroban (0.3 µg/kg/min) while closely monitoring the patient’s clinical status, platelet counts, and coagulation indicators. The dose was then gradually increased to a maximum of 0.75 µg/kg/min. On the 11th day of argatroban therapy, the patient’s platelet count had risen to 126.00 × 10^9^/L, and D-dimer levels had significantly decreased to 2.28 mg/L. Argatroban was discontinued, and oral rivaroxaban was started as sequential anticoagulant therapy (see Fig. 4 for the entire treatment process). On the 19th day after HIT diagnosis, the HIT antibody (HIT-Ab) test was positive (8.5 U/mL), confirming the diagnosis of HIT.

## 3. Discussion

COVID-19 often leads to coagulation disorders, resulting in venous and arterial thromboembolic events. Therefore, anticoagulant therapy is commonly administered to patients with severe COVID-19. In 2021, researchers proposed that for patients with non-severe COVID-19, prophylactic low-dose heparin should be used to reduce the risk of thromboembolic events and associated mortality, provided there are no contraindications.^[9]^ Clinical reports of HIT in patients with mild-to-moderate COVID-19 are rare. At the end of 2023, a case was reported in Japan involving a patient with a history of diabetes who developed severe COVID-19 pneumonia. The patient, owing to elevated D-dimer levels, received heparin treatment, and 13 days later, developed acute coronary syndrome and HIT.^[10]^ In the case reported here, the patient had no underlying diseases and developed pulmonary embolism after the COVID-19 nucleic acid test was negative, suggesting that the thrombotic event may have been triggered by COVID-19 infection. Anticoagulation with enoxaparin led to the development of HIT.

Although HIT can occur with any type or dose of heparin exposure, the risk is influenced by several factors, including the type of heparin used, duration of exposure, route of administration (intravenous/subcutaneous use, extracorporeal circulation, various external devices, and heparin flushes or locks), dosage and treatment strategy, patient demographics, systemic inflammation, level of trauma, and gender. The risk of HIT is higher with unfractionated heparin (UFH) than with LMWH. Surgical patients generally have a higher risk of HIT than medical patients and female patients have a higher risk than male patients. HIT is more likely to occur with therapeutic doses of intravenous heparin than with prophylactic doses administered subcutaneously. The risk of HIT is the lowest when heparin is used for line sealing.^[11,12]^ Andreescu et al demonstrated that in the general inpatient population, the risk of HIT significantly increased after more than 4 days of heparin use (1.2% vs 0.2%).^[13]^

The diagnosis of HIT is primarily based on the patient’s history and clinical manifestations, such as a history of or current heparin use, a significant decrease in platelet count, and the presence or absence of thromboembolic complications. Clinically, the 4Ts scoring system is commonly used to predict the likelihood of HIT occurrence. For patients with a moderate or high probability of HIT, further testing for HIT antibodies can assist in confirming the diagnosis. HIT antibody testing includes mixed antibody (IgG, IgA, IgM) and IgG-specific antibody testing. Mixed antibody testing has lower specificity but higher sensitivity and is primarily used for exclusion purposes. A negative HIT antibody test result can rule out HIT. For patients with a moderate clinical probability, a positive IgG-specific antibody test can confirm the diagnosis; for those with a high clinical probability, a positive IgG-specific antibody test can definitively confirm HIT.^[11]^

For patients with a high suspicion of HIT or those diagnosed with HIT, heparin should be immediately discontinued and non-heparin anticoagulants should be initiated. HIT treatment consists of 2 phases: initial treatment and maintenance therapy. Initial anticoagulation therapy typically involves intravenous agents such as bivalirudin, argatroban, and fondaparinux, whereas maintenance therapy often includes warfarin. Recently, novel oral anticoagulants, such as rivaroxaban, have gained broader use due to their rapid onset, short half-life, fewer drug interactions, and reduced need for frequent monitoring of coagulation parameters. Several small-scale studies abroad have explored the use of rivaroxaban as either an initial or maintenance anticoagulation therapy for HIT, showing promising clinical efficacy.^[14–16]^ In domestic research, Jiang et al retrospectively analyzed clinical data from 12 HIT patients, all of whom had peripheral vascular disease and developed HIT during heparin therapy. After the discontinuation of heparin, all patients were treated with oral rivaroxaban. The study observed complete normalization of platelet counts in all patients, with recovery times ranging from 5 to 9 days (average of 6.8 ± 1.3 days). No active bleeding or new thrombus formation occurred, and the platelet counts remained stable during an average follow-up of 12 ± 5 months.^[17]^ Similarly, Qi et al collected medical records from 21 patients who developed HIT while being treated for deep vein thrombosis with heparin. They analyzed the clinical efficacy and safety of switching to rivaroxaban. The results indicated that D-dimer levels increased and platelet counts decreased after the onset of HIT. However, following rivaroxaban treatment, D-dimer levels gradually decreased and platelet counts increased and normalized. During follow-up (6–12 months), all 21 patients had normal platelet counts, and there were no extensions or recurrences of deep vein thrombosis, nor were there any major or minor bleeding complications. The complete recanalization rate for deep vein thrombosis was 76% (16/21) and the partial recanalization rate was 24% (5/21).^[18]^ In this case study, for a patient highly suspected of having HIT, initial treatment was started with argatroban. Given the patient’s advanced age (86 years) and high bleeding risk, we used a lower dose (0.3 µg/kg/min) of intravenous argatroban and closely monitored the patient’s clinical status, platelet counts, and D-dimer levels. The dose was gradually increased, and argatroban infusion was discontinued once the platelet counts returned to normal. The maximum dose of argatroban used was 0.75 µg/kg/min, which is below the recommended dose in the prescribing information. Rivaroxaban was chosen for subsequent maintenance anticoagulation, and no bleeding or other complications were observed during the clinical follow-up.

It is well known that HIT is associated with a hypercoagulable state. The consensus in the field is that non-heparin anticoagulants should be used for HIT management; however, the bleeding risks associated with these alternatives are challenging to quantify and monitor. This issue is particularly pressing for elderly patients, those with critical illnesses, or individuals with liver or kidney dysfunction, where severe bleeding complications can have dire consequences. Barocas et al proposed that evaluating certain biomarkers may help to assess the hypercoagulability associated with HIT. This approach could aid clinicians in identifying patients with HIT who are at higher risk of thrombotic events. By doing so, it may be possible to mitigate the bleeding risks associated with anticoagulant therapy, ensuring that the treatment is both effective and safe for at-risk patients.^[19]^

## 4. Conclusion

In summary, the COVID-19 virus has continued to mutate, leading to increased transmissibility and decreased mortality rates. As of February 2023, the predominant strain in Beijing was the JN.1 variant, which mostly resulted in mild-to-moderate cases. It is important to note that.

(1) Thrombosis risk post-recovery: Even after a COVID-19 patient’s nucleic acid tests become negative, there remains a risk of venous thromboembolism due to the virus. Clinicians should be vigilant of this potential complication.
(2) Monitoring for HIT: During anticoagulant therapy, HIT should be considered if patients experience a drop in platelet count. For those at-risk for HIT, the 4T scoring system should be used for the initial assessment. HIT antibodies can be tested to confirm diagnosis and reduce the incidence of severe HIT.
(3) Managing bleeding risk: For patients at high risk of bleeding, starting argatroban at a lower dose and gradually increasing it while closely monitoring its clinical efficacy and bleeding risks can be beneficial. Effective treatment can be achieved, even if the dose is below the maximum recommended by the manufacturer.
(4) Future directions: Future approaches may include the use of specific biomarkers to identify patients with HIT who are at a higher risk for thrombotic events. This strategy could help minimize the bleeding risks associated with anticoagulant therapy.

In the current context of coexisting with COVID-19, it is important for clinicians to focus on the risk of venous thromboembolism (VTE) in patients recovering from COVID-19, as well as the risks of HIT and bleeding associated with anticoagulant therapy, along with their management. This is of significant importance for patient clinical diagnosis and treatment.

## Author contributions

**Data curation:** Yu Zhang.

**Formal analysis:** Yu Zhang, Zhenling Chen.

**Investigation:** Yu Zhang.

**Methodology:** Yu Zhang, Zhenling Chen.

**Project administration:** Yi Liu, Zhenling Chen.

**Resources:** Zhenling Chen, Jianying Li, Xuejing Wang.

**Supervision:** Yi Liu.

**Writing – original draft:** Yu Zhang.

**Writing – review & editing:** Yi Liu, Yu Zhang, Zhenling Chen.
